# Glycosphingolipids in Filamentous Fungi: Biological Roles and Potential Applications in Cosmetics and Health Foods

**DOI:** 10.3389/fmicb.2021.690211

**Published:** 2021-07-22

**Authors:** Chunmiao Jiang, Jinxin Ge, Bin He, Bin Zeng

**Affiliations:** ^1^Jiangxi Key Laboratory of Bioprocess Engineering and Co-Innovation Center for In-Vitro Diagnostic Reagents and Devices of Jiangxi Province, College of Life Sciences, Jiangxi Science and Technology Normal University, Nanchang, China; ^2^College of Pharmacy, Shenzhen Technology University, Shenzhen, China

**Keywords:** filamentous fungi, *Aspergillus*, glycosphingolipids, biosynthetic pathway, glucosylceramide and galactosylceramide, application of glycosylceramide

## Abstract

Filamentous fungi are a group of economically important fungi used in the production of fermented foods, industrial enzymes, and secondary metabolites. Glycosphingolipids (GSLs) as constituents of lipid rafts are involved in growth, differentiation, and response to environment stress in filamentous fungi. In addition to these key roles, GSLs are also important in the barrier function of skin to retain moisture as a moisturizing ingredient in cosmetics or health products for their strong biological activity as a functional component. GSLs found in filamentous fungi are divided in two major classes: neutral GSLs (glycosylceramides), glucosylceramides (GlcCers), and/or galactosylceramides (GalCers) and acidic GSLs, mannosylinositol phosphorylceramide (MIPC) and mannosyldiinositol phosphorylceramide [M(IP)_2_C]. Glycosylceramides are one of the abundant GSLs in *Aspergillus* and known to improve skin-barrier function and prevent intestinal impairment as a prebiotic. Some filamentous fungi of *Aspergillus* spp., synthesizing both GlcCer and GalCer, would be an amenable source to exploit glycosylceramides that wildly adding in cosmetics as moisturizing ingredients or health food as dietary supplements. In this minireview, the types, structures, and biosynthetic pathways of GSLs in filamentous fungi, and the relevance of GSLs in fungal growth, spore formation, and environmental stress response are explained. Furthermore, the advantage, potential development, and application of GlcCer and GalCer from filamentous fungi *Aspergillus* spp. are also investigate based on the use of plant GlcCer in health foods and cosmetics.

## Introduction

Filamentous fungi, particularly *Aspergillus* spp., *Trichoderma reesei*, and *Neurospora crassa*, are a group of economically important fungi used in the production of fermented foods, industrial enzymes, antibiotic substances, and organic acids (Brunt, 1986; Cherry and Fidantsef, 2003; Allgaier et al., 2009; Karaffa and Kubicek, 2019). For more than a century, filamentous fungi have been known to produce and secrete different types of enzymes in large quantities, which has resulted in an increased interest in studying them and using them in industrial applications. The production of more than 60% of total industrial enzymes is done by *Aspergillus* genus of filamentous fungi. In addition, filamentous fungi are well-known producers of secondary metabolites with various biological activities. Many of these compounds such as penicillin, cyclosporine or lovastatin are of great importance to human health (Nützmann et al., 2012). For example, two important antibiotics cephalosporin and penicillin are produced by *Cephalosporium acremonium* and *Penicillium chrysogenum*, respectively (Nash and Huber, 1971; Nielsen and Jorgensen, 1995). Furthermore, filamentous fungi are also widely used in the production of organic acids in industrial fermentation, such as citric acid, itaconic acid, fumaric acid, and malic acid (Papagianni, 2007; Knuf et al., 2013; Hu et al., 2014; Karaffa and Kubicek, 2019). *Aspergillus niger* and *Aspergillus oryzae* have a long history of use in the fermentation industry and are generally recognized as safe (GRAS) in accordance with the Food and Drug Administration (FDA). Amylase, a well-known enzyme is produced from *A*. *niger* and *A*. *oryzae* and applicated in diverse processes, ranging from food and beverage to medical (Wirsel et al., 2010; Silano et al., 2018). Soy sauce, a traditional fermented condiment widely consumed in China, Japan, Korea, and other Asian countries, is fermented from soybean by *A*. *oryzae* (Liang et al., 2009). Therefore, due to the economic importance of filamentous fungi, the secondary metabolite pathways, organic compound, and fermentation processes in these filamentous fungi have attracted attention of the scientists.

In the industrial application of filamentous fungi, the conditions of the fermentation play a vital role in the growth and metabolism of a microbial population. Filamentous fungi have the potential to grow or ferment under diverse environmental conditions by utilizing a wide variety of substrates as nutrients (Chen et al., 2011). The ability of microorganisms to adapt to different environmental factors has attracted considerable attention, with many studies investigating the molecular mechanisms of microorganism in response to environmental stress. Fungi adapt to environmental changes using a range of molecular mechanisms. One mechanism is *via* change in the composition of cellular lipids, such as phospholipids, neutral lipids, or unique glycosphingolipids (GSLs) (Suutari, 1995; Řezanka et al., 2016, 2018). Fungal GSLs, including neutral and acidic GSLs, are the main lipid components of microdomains in fungal membranes and are clustered along with sterols to form lipid rafts, which play crucial roles in cell polarization, hyphal growth, fungal fitness, and adaptation to most diverse environments as a signaling molecules (Sonnino et al., 2007; Guimarães et al., 2014; Řezanka et al., 2016, 2018). Although GSLs have been studied extensively, the details of their function are difficult to understand owing to complex and dynamic changing synthetic pathways of interconversion and utilization. Nonetheless, the role of yeast GSLs in response to heat stress has been investigated thoroughly (Chen et al., 2013; Řezanka et al., 2016, 2018). However, the GSL pathways and related genes that contribute to fungal growth, differentiation, morphogenesis, particularly those involved in response to environmental stress in filamentous fungi, are less appreciated.

In addition to fungal growth and response to environmental stress, neutral GSLs (also call glycosylceramides) are also known to have nutritive functions such as preventing intestinal impairment and enhancing the moisture content of skin (Ono et al., 2010; Duan et al., 2012; Hamajima et al., 2016). For example, neutral GSLs can be added to food as a “functional components” for their strong biological activity in health food products such as nutritional supplements, infant foods, and beverages that help in reduction of blood pressure, activation of immunity and inhibition of cancer cell proliferation. Previous studies reported that *A*. *oryzae* glycosylceramides functions as a prebiotic, can alter the intestinal microbial flora and increase *Blautia coccoides* (Hamajima et al., 2019). Since there are many reports of the effects of *B*. *coccoides* on health, an increase in intestinal *B*. *coccoides* by *koji* glycosylceramide might be the connection between intestinal microbial flora and healthy. Besides, neutral GSLs are also important due to their barrier function as a moisturizing ingredient in cosmetics that help of skin retain its moisture (Alessandrini et al., 2004; Tessema et al., 2017; Miyagawa et al., 2019). However, plant cell membrane contents such as neutral GSLs, are difficult to extract due to the thick cell wall and GSLs from neural tissues of animals are not acceptable for cosmetic or other human use due to the underlying risk of prion diseases. Therefore, filamentous fungi *koji*, which are safe for humans and contain abundant (0.5–2 mg/g dry weight koji) neutral GSLs, would be important resources for exploiting neutral GSLs in the future.

In this review, we discuss the relevance of GSLs in fungal growth, spore formation and environmental stress response, which are key to the production of fermented foods, commercial enzymes, and secondary metabolites in industrial fermentation, and the potential development and application of filamentous fungi GSLs in cosmetics and health foods.

## The Kinds and Structures of GSLs in Filamentous Fungi

**FIGURE 1:**
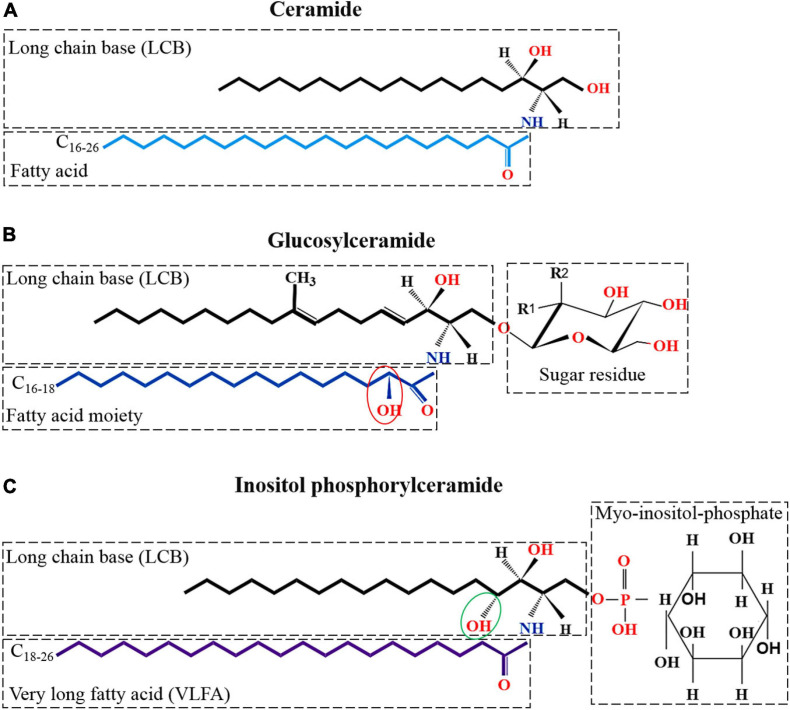
Kinds and structures of glycosphingolipid in filamentous fungi. **(A)** Structure of ceramide; the basic unit of neutral and acidic glycosphingolipids; made up of a LCBs and a FA moiety. **(B)** Structure of neutral glycosphingolipid which is linked to a sugar residue on the base of ceramide. Neutral glycosphingolipid contains an-OH moiety at C_2_ in the FA chain (showed with red ellipse); a double bond between C_4__–__5_ and C_8__–__9_; and a methyl C_9_ of the LCB. Monosaccharide may be either glucosyl (Glc) (R1 = OH, R2 = H) or galactosyl (Gal) residues (R1 = H, R2 = OH). **(C)** The basic structure of acidic glycosphingolipid, inositol phosphorylceramide, is shown. Inositol phosphorylceramide is different from neutral glycosphingolipids in that it contains an additional hydroxy at C_4_ of the LCB (showed with green ellipse) and lacks double bonds between C_4__–__5_ and C_8__–__9_, and the C_9_ methyl of the LCB. In addition, acidic glycosphingolipids are composed of a very long FA chain (C_18__–__26_) instead of the C_16__–__18_ chain that found in neutral glycosphingolipids.

Glycosphingolipids are ubiquitous membrane components and are involved in many biological processes crucial for filamentous fungi, such as growth, signal transduction, morphological transition, and pathogenesis (Heung et al., 2006; Huber et al., 2019). Our knowledge of GSL biosynthesis in filamentous fungi is mainly based on investigations of yeasts, the human pathogenic *Candida albicans* (Oura and Kajiwara, 2010), the model organisms *N. crassa* (Park et al., 2005) and *Aspergillus nidulans* (Levery et al., 2002; Fernandes et al., 2016), and the plant pathogen *Fusarium graminearum* (Duarte et al., 1998; Ramamoorthy et al., 2007, 2009; Zaüner et al., 2008). The basic structure of GSL consists of an 18-carbon-alcohol chain called long chain base (LCB) that is linked to a C_16__–__26_ fatty acid (FA) chain through an amide bond to form ceramide (Figure 1A), which in turn is linked to a polar head group (Figures 1B,C) (Barreto-Bergter et al., 2004, 2011; Del Poeta et al., 2014). GSLs have been isolated and identified from various filamentous fungi, such as *C. albicans*, *F. graminearum*, *N. crassa*, and *Aspergillus* spp., and divided in two major classes of neutral and acidic GSLs (Totani et al., 2001; Park et al., 2005; Zhang et al., 2007; Zaüner et al., 2008; Takahashi et al., 2009; Hirata et al., 2012; Guimarães et al., 2014; Tani et al., 2014; Fernandes et al., 2016).

In filamentous fungi, neutral GSLs (also call glycosylceramides) include glucosylceramide (GlcCer) and galactosylceramide (GalCer), while acidic GSLs consist of two forms of glycosyl inositol phosphoryl ceramides (GIPCs), mannosylinositol phosphorylceramide (MIPC) and mannosyldiinositol phosphorylceramide [M(IP)_2_C] (Barreto-Bergter et al., 2004, 2011; Buré et al., 2014). Neutral GSLs contain 9-methyl-Δ4,Δ8-sphingadienine as the LCB linked to a C_16__–__18_ N-2-hydroxy or unsaturated N-2-hydroxy-Δ3 FA chain and a glucose or galactose moiety to form GlcCer or GalCer. In contrast, the ceramide moiety of acidic GSLs is composed of a 4-hydroxysphinganine (phytosphingosine) as the LCB attached to a very long fatty acid (VLFA) chain (C_18__–__26_) and complex glycan groups *via* an inositol phosphate linker. All filamentous fungi investigated so far contain GlcCer or GalCer, with most mold species, such as *Aspergillus fumigatus* (Boas et al., 1994; Toledo et al., 1999; Fontaine, 2017), *A. niger* (Wagner and Fiegert, 1969; Levery et al., 2000), *A. oryzae*, *Aspergillus sojae*, and *Aspergillus awamori* (Tani et al., 2014), containing both GlcCer and GalCer (Table 1). Interestingly, *Sporothrix schenckii* mycelia synthesize only GlcCer, while the yeast forms are found to contain both GlcCer and GalCer (Table 1; Dickson and Lester, 1999; Toledo et al., 2000). In fungi, GlcCer and GalCer are considered the final step of the pathway, whereas in mammalian cells GlcCer and GalCer are then used to make hundreds of complex GSLs. In fungi and plants, acidic GSLs include inositol phosphorylceramides (IPCs), which are used as building blocks for more complex molecules, such as MIPC and M(IP)_2_C. In summary, GlcCer is the only GSLs found in all organisms studied, especially eukaryotic cell, such as fungi, plants as well as invertebrates and vertebrates (Table 2). In contrast, GIPCs occur only in fungi and plants, whereas GalCer is restricted to fungi, vertebrates, and invertebrates (Table 2).

**TABLE 1:** The type and structure of neutral GSLs in filamentous fungi.

Organism	GlcCer	GalCer	Structural features of FA	Structural features of LCB	References
*Aspergillus fumigatus*	+	+	2-hydroxyoctadec-Δ3-unsaturation	Δ4, Δ8-unsaturation; 9-methyl	Boas et al., 1994; Toledo et al., 1999; Fontaine, 2017
*Aspergillus niger*	+	+	2-hydroxyoctadec-Δ3-unsaturation	Δ4, Δ8-unsaturation; 9-methyl	Wagner and Fiegert, 1969; Levery et al., 2000
*Aspergillus nidulans*	+	−	2-hydroxyoctadec	Δ4, Δ8-unsaturation; 9-methyl	Levery et al., 2002
*Aspergillus oryzae*	+	+	2-hydroxyoctadec	Δ4, Δ8-unsaturation; 9-methyl	Fujino and Ohnishi, 2003; Tani et al., 2014
*Aspergillus versicolor*	+	−	2-hydroxyoctadec	Δ4, Δ8-unsaturation; 9-methyl	Boas et al., 1994
*Aspergillus sojae*	+	+	2-hydroxyoctadec	Δ4, Δ8-unsaturation; 9-methyl	Tani et al., 2014
*Aspergillus awamori*	+	+	2-hydroxyoctadec	Δ4, Δ8-unsaturation; 9-methyl	Tani et al., 2014
*Aspergillus kawachii*	+	−	2-hydroxyoctadec	Δ4, Δ8-unsaturation; 9-methyl	Hirata et al., 2012
*Sporothrix schenckii* (mycelia forms)	+	−	2-hydroxyoctadec-Δ3-unsaturation	Δ4, Δ8-unsaturation; 9-methyl	Dickson and Lester, 1999; Toledo et al., 2000
*Sporothrix schenckii* (yeast forms)	+	+	2-hydroxyoctadec-Δ3-unsaturation	Δ4, Δ8-unsaturation; 9-methyl	Dickson and Lester, 1999; Toledo et al., 2000

*GlcCer, glucosylceramide; GalCer, galactosylceramide; FA, fatty acid; LCB, long chain base. +, presence; −, absence.*

**TABLE 2:** Relative presence of glycosphingolipids (GSLs) in filamentous fungi, plants, invertebrates, and vertebrates.

Kinds of GSLs	Filamentous fungi	Plants	Invertebrates and vertebrates
GlcCer	+	+	+
GalCer	+	−	+
MIPC	+	+	−
M(IP)_2_C	+	+	−

*+, presence; −, absence; GlcCer, glucosylceramide; GalCer, galactosylceramide; MIPC, mannosylinositol phosphorylceramide; M(IP)_2_C, mannosyldiinositol phosphorylceramide.*

Neutral and acidic GSLs have specific differences in the structures of their ceramide backbones. In filamentous fungi, the differences in hydroxylation, saturation levels and methylation levels result in different LCBs of both GSLs (Warnecke and Heinz, 2003; Gault et al., 2010). Neutral GSLs normally contain the monosaccharide, glucose or galactose, in glycosidic linkage with the 9-methyl-Δ4, Δ8-sphingoid (ceramide) (Takahashi et al., 2009). The structure of 9-methyl-Δ4, Δ8-sphingadienine, which contains two double bonds in C_4__–__5_ and C_8__–__9_ and a methylation at C_9_ (Fujino and Ohnishi, 1977; Barreto-Bergter et al., 2004, 2011; Takahashi et al., 2009; Del Poeta et al., 2014), is unique to neutral GSLs of filamentous fungi and distinguishes them from plant and mammalian sphingosines (Figure 1B). While Δ4-sphingenine with a double bond in C_4__–__5_ is predominantly found as LCB in mammals and is rare in fungi and plants, Δ4,Δ8-sphingadienine with two double bonds in C_4__–__5_ and C_8__–__9_ is found in plants (Sperling and Heinz, 2003; Barreto-Bergter et al., 2004; Takahashi et al., 2009). In contrast, acidic GSLs are different from neutral GSLs in that they contain an additional hydroxyl group at C_4_ of their LCB (4-hydroxyshinganine) (Figure 1C showed with green ellipse) and lack Δ4,Δ8-unsaturations and C_9_-methylation found in the LCBs of neutral GSLs in filamentous fungi (Figure 1C). In addition, the FA lengths of neutral and acidic GSLs is also different. In filamentous fungi, C_16__–__18_ FA chains are observed in neutral GSLs, and C_24__–__26_ chains are observed in acidic GSLs (Marques et al., 2018), while the mammalian FA length is predominantly a C_16_ or C_18_ FA chain. In plants, the lengths of FA chains range from C_14_ to C_26_ (Sperling and Heinz, 2003). Interestingly, some filamentous fungal GSLs contain a Δ3-desaturated in FA chain, which is a unique modification of fungal GSLs and has been reported in *A*. *fumigatus* (Boas et al., 1994; Toledo et al., 1999), *A*. *niger* (Wagner and Fiegert, 1969; Levery et al., 2000), and *S. schenckii* (Toledo et al., 2001; Table 1).

In summary, filamentous fungal GlcCer or GalCer mainly consists of a β-D-glucose or galactose attached to C_1_ of 9-methyl-Δ4,Δ8-sphingadienine which is N-acylated with a C_16__–__18_ N-2-hydroxy or N-2-hydroxy-Δ3 FA chain. In contrast, the ceramide moiety of acidic GSLs is usually formed by 4-hydroxysphinganine (phytosphingosine) as the LCB, bound to a VFFA chain (C_18__–__26_), linked to a polar head group. IPC is further modified upon addition of mannose and a second inositol phosphate group to generate MIPC (with a mannose sugar unit) and M(IP)_2_C (with two inositol groups).

## Key Genes and Important Products Involved in Biosynthesis of GSLs in Filamentous Fungi

**FIGURE 2:**
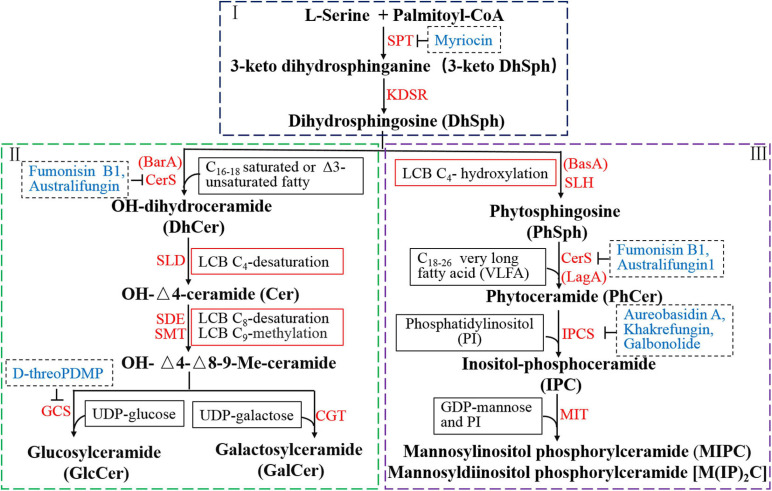
Reorganized biosynthetic pathway of glycosphingolipid in filamentous fungi. The first part (I) involves two key enzyme-catalyzed reactions and is the starting point and common to neutral and acidic GSL synthesis. The second part (II) represents the biosynthetic pathway of neutral GSLs (GlcCer and GalCer) from DhSph including four enzyme-catalyzed reactions. GlcCer and GalCer are the final products of neutral GSL pathway in filamentous fungi. The third part (III) is the biosynthetic process of acidic GSLs, including the production of IPC, MIPC, and M(IP)_2_C, which also starts from DhSph and includes three enzyme-catalyzed reactions. IPCs, used as building blocks for more complex molecules, are further modified upon addition of mannose and a second inositol phosphate group to generate MIPC and M(IP)_2_C. Red texts represent genes or relative enzymes involved in the GSL pathway. Blue texts indicate the inhibitors of the specific enzyme in the biosynthetic steps.

Raw ceramide for the synthesis of GSLs is derived from three pathways, de novo GSL synthesis, sphingomyelin degradation and GSL recycling. Here we focus on the de novo synthesis pathway of GSLs that is conserved among filamentous fungi (Gault et al., 2010). The whole process of GSL synthesis pathway was divided into three modules for better understanding, the synthesis of dihydrosphingosine, synthesis of neutral GSLs, and synthesis of acidic GSLs (Figure 2). In this section, the productions of GSLs *via* the *de novo* biosynthetic pathway and the key genes involved are discussed.

### The Role of Dihydrosphingosine

The biosynthesis of dihydrosphingosine (DhSph), involves two key enzyme-catalyzed reactions and is the starting point in the biosynthesis of all GSLs (Figure 2I). The first and rate-determining step is the condensation of palmitoyl coenzyme A (palmitoyl-CoA) and L-serine, catalyzed by serine palmitoyltransferase (SPT), to produce 3-keto dihydrosphingosine (3-keto DhSph), which is then reduced to DhSph by 3-keto DhSph reductase (KDSR) (Barreto-Bergter et al., 2004). The genes encoding SPT and KDSR are conserved among all organisms and have been identified from various species of fungi. Two genes, *LCB1* and *LCB2*, which are necessary for SPT activity, have been identified in *Saccharomyces cerevisiae* (Buede et al., 1991; Nagiec et al., 1994). Deletion of either *LCB1* or *LCB2* was found to abolish GSL production in yeast (Mukherjee and Dekker, 1990; Zhao et al., 1994; Dickson, 2008). In *A*. *nidulans*, the gene encoding SPT was identified and named *lcbA* owing to its homology to the *S*. *cerevisiae LCB1*. The *lcbA* is essential for polarity and growth in *A. nidulans* and under the control of the *alcA* promoter, which is alcohol inducible and glucose repressible (Waring et al., 1989; Cheng et al., 2001). An important third subunit of SPT is Tsc3p, which coimmunoprecipitates with LCB1 or/and LCB2, and is required for optimal and high level SPT activity during maximal GSL biosynthesis in *S. cerevisiae* (Gable et al., 2000).

The role of 3-keto DhSph reductase remains poorly investigated in filamentous fungi. In *A*. *fumigatus*, KDSR is encoded by the *ksrA* gene, but the biological role of *ksrA* in *Aspergillus* remains to be elucidated (Fornarotto et al., 2006). Deletion of *C*. *albicans ksr1* shows compromised filamentation, suggesting that the expression of *ksr1* may be important for polarized growth (Fornarotto et al., 2006).

Although poorly studied, the production of DhSph is a critical and common pathway in the biosynthesis of neutral and acidic GSLs. This compound can generate two distinct ceramide pools of dihydroceramide and phytoceramide, which are utilized for the formation of neutral and acidic GSLs, respectively. Two genes, *BarA* and *BasA*, are involved in this step in filamentous fungi (Li et al., 2006; Rittenour et al., 2011; Cheon et al., 2012; Fontaine, 2017). The ceramide pool involved in the neutral GSL synthesis (dihydroceramide) is generated by *BarA*, while acidic GSL synthesis (phytoceramide) is catalyzed by *BasA* (also called *Sur2* in *S. cerevisiae*). Therefore, filamentous fungi possess two distinct ceramide pools that make independent contributions to polarized hyphal growth, through the formation of specialized lipid microdomains that regulate the organization of the cytoskeleton (Li et al., 2006).

### Biosynthetic Process of Neutral GSLs

Synthesis of the neutral GlcCer and GalCer from DhSph includes four enzyme-catalyzed reactions (Figure 2II).

#### OH-Dihydroceramide

Dihydrosphingosine is first N-acylated with saturated or Δ3-unsaturated C_16__–__18_ FA chain catalyzed by ceramide synthase (CerS) to produce dihydroceramide (DhCer). A hydroxyl group is then inserted at C_2_ in the FA chain of DhCer generating OH-DhCer. The ceramide pool involved in the DhCer synthesis is unique to filamentous fungi and catalyzed by ceramide synthase encoded by *BarA* (or *Cer1*, *Bar1*) (Li et al., 2006; Rittenour et al., 2011). *BarA* is specifically necessary to produce neutral GSLs in fungi and contributes differentially to polarized hyphal growth. The Δ*BarA* or Δ*Cer1* or Δ*Bar1*mutant can produce normal IPC but completely lacks GlcCer, fails to display the distinct sterol-rich domain at the hyphal tip and is incapable of producing perithecia (Rittenour et al., 2011; Cheon et al., 2012; Munshi et al., 2018).

#### OH-Δ4-Ceramide

The LCB of OH-DhCer is reduced by sphingolipid Δ4-desaturase (SLD) to generate OH-Δ4-ceramide in the cytosolic face of the endoplasmic reticulum (ER) (Michel and van Echten-Deckert, 1997; Ternes et al., 2002). Sphingolipid Δ4-desaturase, encoded by the *DEGS1*, is involved in the double bond formation at C_4__–__5_ of DhCer (Michel et al., 1997; Rodriguez-Cuenca et al., 2015).

#### 9-Methyl-Δ4, Δ8-Ceramide

Then, a double bond at C_8__–__9_ and a methyl group at C_9_ are introduced into the LCB by sphingolipid Δ8-desaturase (SDE) and sphingolipid C_9_-methyltransferase (SMT), respectively, resulting in the formation of OH-Δ4,Δ8-9-methyl-ceramide (Ternes et al., 2006; Rhome et al., 2007). Sphingolipid Δ8-desaturase is encoded by *SdeA* in *A*. *nidulans*. There are two genes encoding C_9_-methyltransferases in *A*. *nidulans* (*smtA* and *smtB*) and *F. graminearum* (*FgMT1* and *FgMT2*), but only one candidate has been identified in *N. crassa* (Ternes et al., 2006). OH-Δ4,Δ8-9-methyl-ceramide is produced in the ER and transported to the Golgi apparatus for synthesis of GalCer and GlcCer.

#### GlcCer and GalCer

The final step of the neutral GSL biosynthetic pathway is the transfer of a sugar residue from UDP-glucose or UDP-galactose to the OH-Δ4,Δ8-9-methyl-ceramide catalyzed by glucosylceramide synthase (GCS) or ceramide galactosyltransferase (CGT), respectively in the Golgi apparatus (Leipelt et al., 2001; Warnecke and Heinz, 2003; Ternes et al., 2011). GCS is encoded by *GCSA* or *GCS1* that is essential for mycelial growth and filamentation in filamentous fungi (Ramamoorthy et al., 2007; Fernandes et al., 2016). Although most of the genes responsible for GlcCer biosynthesis have been identified and cloned, the gene encoding ceramide galactosyltransferase has been detected only in *N*. *crassa* and *Magnaporthe grisea* (Lester et al., 1974; Maciel et al., 2002). Limited information is available on the occurrence of GalCer in fungi and information on their function or on the galactosyltransferase responsible for their formation is lacking.

### Synthetic Process of Acidic GSLs

The synthesis of acidic GSLs, including the productions of IPCs, MIPCs, and M(IP)_2_Cs, also starts from DhSph and includes three enzyme-catalyzed reactions (Figure 2III).

#### Phytosphingosine

The LCB at C_4_ of DhSph is hydroxylated by the sphingolipid C_4_-hydroxylase (SLH) that is present only in fungi and plants. This reaction generates phytosphingosine (PhSph) that is essential for filamentous fungal growth and viability (Li et al., 2006). In *S*. *cerevisiae*, *Sur2* encoding sphingolipid C_4_-hydroxylase is essential for sphingolipid C_4_-hydroxylation activity but is not essential for normal growth (Cliften et al., 1996; Haak et al., 1997). In *A*. *nidulans*, deletion of *basA* leads to a reduced growth with an hyperbranching of hyphae, an aberrant cell wall thickening and a strong defect in conidiation (Li et al., 2006, 2007).

#### Phytoceramide

A C_18__–__26_ FA chain is linked to PhSph *via* an amide by ceramide synthase (CerS) to form phytoceramide (PhCer) that seems to be relevant for fungal viability and hyphal morphogenesis. In *A*. *nidulans*, ceramide synthase is encoded by the *lagA* and is required for the apical growth and morphology (Li et al., 2006). PhCer is transported from the ER to the outer leaflet of the Golgi membrane for the synthesis of IPC in the next step (Dickson et al., 2006).

#### IPC, MIPC, and M(IP)_2_C

The myo-inositol-1-phosphate group is transferred from phosphatidylinositol (PI) to the C_1_ hydroxyl of PhCer to produce IPC, catalyzed by the IPC synthase (Cheng et al., 2001; Dickson et al., 2006). IPC synthase is also a rate-limiting enzyme encoded by the *IPC1* or *AUR1*, found in *S*. *cerevisiae*, *Schizosaccharomyces pombe*, *Candida* spp., and *Aspergillus* spp. (Hashida-Okado et al., 1996; Nagiec et al., 1997; Kuroda et al., 1999; Georgopapadakou, 2000; Cheng et al., 2001). The *aurA* is required for polarized cell growth in *A*. *nidulans*; repression of *aurA* in *A*. *nidulans alcA*::*aurA* causes a terminal phenotype of germinating spores and lacks polarized hyphal growth (Cheng et al., 2001). In filamentous fungi, IPC is further modified by addition of mannose and a second inositol phosphate group in a reaction catalyzed by MIPC transferase (MIT, encoded by *mitA*) to generate two products, MIPC that has a mannose sugar unit and M(IP)_2_C that has a mannose unit as well as two inositol groups (Leber et al., 1997). The *mitA* gene is essential for the addition of the first mannose residue to the inositol ring. Although *A*. *fumigatus* Δ*mitA* abolishes the production of MIPCs and MIPC-derived GSLs, leading to accumulation of the precursor IPC, the Δ*mitA* mutant shows normally growth and no defects in cell wall or membrane organization, suggesting that MIPC is not critical for fungal differentiation (Kotz et al., 2010). MIPC and M(IP)_2_C are two forms of GIPCs found in several fungi that are particularly regulated during morphogenesis (Takahashi et al., 2009; Guimarães et al., 2014; Buré et al., 2014).

### Enzyme Inhibitors in Key Steps of GSL Biosynthesis

Blocking GSL biosynthesis has become a target for developing antifungal therapies and understanding fungal biology. Thus, enzyme inhibitors of synthetic GSLs for a variety of biological processes have already been demonstrated. There are five key steps in which enzymes can be blocked by inhibitors in the GSL biosynthetic pathway (Figure 2 the blue fonts). As the first rate-limiting enzyme of the GSL biosynthetic pathway, SPT has been the subject of many studies to identify inhibitors. One of the earliest SPT inhibitors identified is the mechanism-based inhibitor L-cysteine (Zheng et al., 1994, 1998). The most widely used and studied natural product SPT inhibitors are myriocin and sphingofungin that have been shown to block biofilm formation and polarized growth of fungal hyphae in *Candida* and *Aspergillus* species (Cheng et al., 2001; Lattif et al., 2011; Perdoni et al., 2015; Harrison et al., 2018). In addition, β-chloroalanine, β-fluoroalanine, and halide can also be used to inhibit SPT (Lev and Milford, 1981; Medlock and Merrill, 1988; Smith and Merrill, 1995). Fumonisin B1 and australifungin are inhibitors of ceramide synthase that alter GSL metabolism by inhibiting ceramide synthesis, and act as antifungal agents in *Cryptococcus*, *Candida*, and *Aspergillus* species (Mandala et al., 1995; Merrill et al., 2001). Glucosylceramide synthase is involved in the final step of GlcCer synthesis and can be blocked by D-threo-1-phenyl-2-decanoylamino-3-morpholino-1-propanol (D-threoPDMP) that cause a reduction in hyphal germination and colony growth in *A*. *nidulans*, *A*. *fumigatus*, and *Aspergillus terreus* (Levery et al., 2002).

Inositol phosphorylceramides synthases are unique structures in fungi involved in many cellular processes, including growth, differentiation, and morphogenesis, but are not found in mammals (Barr and Lester, 1984; Levery et al., 1998, 2002; Loureiro y Penha et al., 2001). Therefore, IPC synthases constitute potential targets for the development of new antifungal drugs, as inhibiting it will lead to the accumulation of PhSph and PhCer and, ultimately fungal death. Aureobasidin A, khakrefungin, and galbonolide inhibitors of IPC synthase can block transfer of myo-inositol-1-phosphate from PI to ceramide, resulting in accumulation of PhSph and PhCer, and complete inhibition of IPC production, along with cell cycle arrest in *C*. *albicans*, *Cryptococcus neoformans*, *S*. *cerevisiae*, and *A*. *nidulans* (Cheng et al., 2001; Cerantola et al., 2009; Tan and Tay, 2013). Therefore, IPC inhibitors, with low toxicity in mammals due to the lack of a mammalian IPC synthase, might be possible candidates as ideal antifungal drugs (Takesako et al., 1993; Mandala et al., 1997, 1998; Georgopapadakou, 2000; Rollin-Pinheiro et al., 2016).

## Biological Roles of GSL in Filamentous Fungi

### Role of GSLs in Filamentous Fungal Growth

**FIGURE 3:**
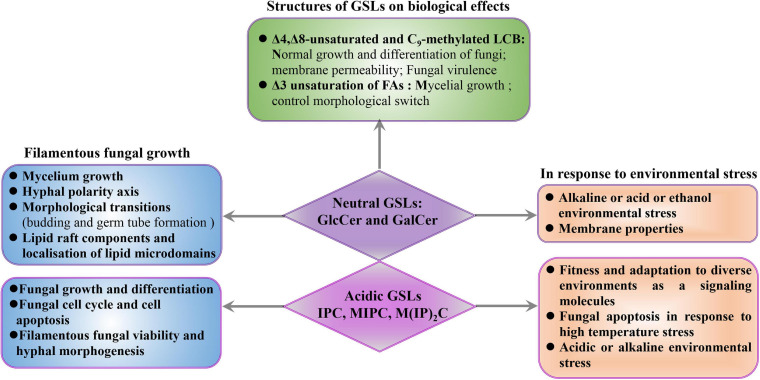
Biological roles of GSLs in filamentous fungi. The effects of GSLs on filamentous fungi were discussed from three aspects, mainly including fungal growth and environmental stress. Meanwhile, the functions of the Δ3-unsaturated FAs, and Δ4, Δ8-unsaturated, and C9-methylated structures of GlcCer and GalCer were also summarized.

Glycosphingolipids are the main lipid components of microdomains and are clustered along with sterols in specialized membrane microdomains to form lipid rafts in filamentous fungi (Sonnino et al., 2007). These regions play crucial roles in cell polarization and hyphal growth, mediated by the cytoskeleton and accumulation of lipid raft components at the growing tip of hyphal cells (Cheng et al., 2001; Barreto-Bergter et al., 2004; Pearson et al., 2004; Nimrichter and Rodrigues, 2011; Guimarães et al., 2014). Biological functions of GSLs have been studied through gene deletion and inhibitory approaches in filamentous fungi (Rittershaus et al., 2006; Oura and Kajiwara, 2008, 2010; Ramamoorthy et al., 2009; Zhu et al., 2014; Fernandes et al., 2016). Several studies have demonstrated that impairment of any steps of GlcCer biosynthetic pathway leads to compromised mycelium growth, hyphal elongation, and lipid raft mislocalization. For example, disruption of the initial step of GlcCer synthesis by deletion of *barA* or by inhibition in presence of HSAF strongly reduces polarized growth and leads to the formation of enlarged and hyperbranched hyphae, with altered cell wall (Li et al., 2006, Li et al., 2009). Similarly, disruption of the final step of GlcCer synthesis by deletion of *GCS* or by glucosylceramide synthase inhibitors also impairs hyphal extension in *A*. *nidulans* and inhibits morphological transitions such as budding and germ tube formation in *A*. *fumigatus* (Levery et al., 2002; Ramamoorthy et al., 2007; Fernandes et al., 2016). These results suggest that GlcCer synthase is essential for the establishment and maintenance of the polarity axis, fungal growth, and differentiation in filamentous fungi (Figure 3).

In filamentous fungi, not only is growth and differentiation reduced upon impairment of neutral GSL synthesis, but growth also relies on the production of IPC (Figure 3). Inhibition of IPC synthase is followed by an accumulation of PhSph and PhCer intermediates that lead to fungal cell cycle arrest and apoptosis (Cheng et al., 2001). Integrity of the IPC synthesis pathway is relevant for filamentous fungal viability, not only due to the role of IPC in fungal differentiation but also due to the need for highly regulation of DhSph, PhSph, and PhCer levels, as DhSph and PhSph are highly toxic to fungal cells. Thus, changes in their levels may have uncontrolled effects on signaling events, resulting in fungal cell death which indicate that PhCer synthase and IPC synthase can be potential targets for development of new antifungal drugs, as inhibiting these enzymes will lead to the accumulation of PhSph and PhCer, and, ultimately, fungal death.

### Role of GSLs in Response to Environmental Stress in Filamentous Fungi

In addition to fungal polarization and hyphal growth, GSLs also play important role in filamentous fungal fitness and adaptation to diverse environments as a signaling molecules (Grimm et al., 2006; Lingwood and Simons, 2010; Figure 3). The fungal heat shock response is characterized by the production of heat shock proteins that function as chaperonins, and in the processes of protein degradation. GSLs possess the capacity to serve as bioactive signaling molecules in early heat stress response. This signaling function influences the regulation of the cell cycle and the synthesis of heat shock proteins, which have important secondary effects, especially if heat shock proteins are not available to serve as protectors of other proteins (Jenkins, 2003). For example, *C. albicans* treatment with inhibitor of SPT, leads to the decrease in lipid domain accumulation, resulting in less recruitment of heat-shock proteins (Insenser et al., 2006). In addition, environmental stresses directly affect the representation of fungal GSL classes (Řezanka et al., 2018). Thermophilic microorganisms can change the contents of IPC, MIPC, or M(IP)_2_C to mediate fungal apoptosis in response to high temperature stress (Řezanka et al., 2016, 2018). *N. crassa* synthesizes PhCer as signal molecules to mediate fungal apoptosis in response to heat stress (Plesofsky et al., 2008). In *S. cerevisiae*, the accumulation of IPC is detrimental to yeast under low pH conditions, and downregulation of IPC levels is one of the adaptation mechanisms for low pH conditions (Otsu et al., 2020). In contrast, reduction of the IPC level increases the sensitivity of *C*. *neoformans* to low pH and impairs the growth and the pathogenicity of *C*. *neoformans* (Luberto, 2001). Besides, blocking the pathway of GSL synthesis or adding exogenous GSLs can significantly affect fungal tolerance to acidic or alkaline environmental stress. For example, in *C*. *neoformans*, the Δ*cer1* mutant cannot survive in acid or alkaline environments due to its inability to synthesize GlcCer (Ishibashi et al., 2012; Munshi et al., 2018). Besides, the *C*. *neoformans*Δ*sld8* mutant that only synthesizes saturated GlcCer is more susceptible to membrane stressors and shows increased membrane permeability, resulting in decreased stress resistance (Raj et al., 2017). Similarly, *S*. *cerevisiae*, which is incapable of synthesizing GlcCer, can adapt to alkaline and ethanol stress after exposure to *Aspergillus kawachii*-derived GlcCer *via* altering the yeast membrane properties by exogenous GlcCer (Sawada et al., 2015). These results demonstrate that GSLs play important roles in response to environmental stress in fungi (Figure 3).

### Structures of GSLs on Biological Effects in Filamentous Fungi

Impairment of polarized hyphal growth and mislocalization of lipid microdomains were observed when the biosynthetic steps of sphingolipid desaturase and sphingolipid C_9_-methyltransferase were interrupted, demonstrating that unsaturation and C_9_-methylation of the neutral GSLs are essential for growth and differentiation in filamentous fungi. The structures of Δ4,Δ8-unsaturated and C_9_-methylated LCB, distinguishing filamentous fungi from plant and mammalian sphingosines, are unique to fungal neutral GSLs (Barreto-Bergter et al., 2004, 2011; Del Poeta et al., 2014). Disruption of the *A*. *nidulans sdeA* leads to increase accumulation of saturated and unmethylated GlcCer and reduce mycelium growth (Fernandes et al., 2016). Previous studies in *C*. *neoformans* showed that Δ*sld8* mutant synthesizing only saturated GlcCer is more susceptible to membrane stressors follow by increasing membrane permeability, even though saturated GlcCer produced more lipid rafts than unsaturated GlcCer species (Raj et al., 2017). The C_9_-methylated found in LCB is required for normal growth and differentiation in filamentous fungi (Ramamoorthy et al., 2009; Fernandes et al., 2016). *A*. *nidulans smtA* deletion combined with conditional repression of *smtB* significantly increases unmethylated GlcCer levels and compromises filamentous growth (Fernandes et al., 2016). The Δ*Fgmt2* mutant produces 65–75% unmethylated GlcCer shows severe growth defects when compared to the wild-type strain (Ramamoorthy et al., 2009). In pathogenic yeasts, deletion of the gene encoding C_9_-methyltransferase results in a mutant with attenuated virulence (Noble et al., 2010; Singh et al., 2012). Interestingly, certain plant defensins require the C_9_-methylation for fungal GSL recognition (Oguro et al., 2014; Fernandes et al., 2016), which suggest that plant defensins may have a therapeutic potential for treatment of fungal infections for fungus specific C_9_-methylation (Figure 3).

The unsaturated FAs of neutral GSLs are also important for the mycelial growth in filamentous fungi. The Δ3-unsaturated FA is a unique modification of fungal GSLs, which has been reported in neutral GSLs of *A. oryzae* (Yasuhiko and Masao, 1997), *A*. *fumigatus* (Toledo et al., 1999), and *S*. *schenckii* (Toledo et al., 2001), and is involved in signaling pathways that control morphological switch (Figure 3). In fact, the ratio of saturated and Δ3-unsaturated 2-hydroxy FAs vary among the GSLs from different fungal morphotypes. For example, only 15% of GlcCer extracted from the *Paracoccidioides brasiliensis* yeasts is composed of Δ3-unsaturated FAs, while 50% of GlcCer contains the Δ3-unsaturation in *P*. *brasiliensis* mycelium (Toledo et al., 1999). Similarly, GlcCer from *Histoplasma capsulatum* yeast contains a higher proportion of saturated FAs, while the GlcCer from mycelium is almost exclusively constituted by the Δ3-unsaturated FAs (Toledo et al., 2001). The higher contents of Δ3-unsaturated GlcCer in mycelial forms of *P*. *brasiliensis* and *H*. *capsulatum* may be ascribed to the activation of desaturase activity during the yeast-to-hypha transition, suggesting that Δ3-unsaturation of GlcCer may be involved in signaling pathways that control morphological switch.

In contrast to GlcCer, most studies of GalCer have been performed in mammals and its biological function in filamentous fungi remains unknown. It has been previously shown that sphingolipids regulate the activity of protein kinases, such as protein kinase C and protein phosphatases, involved in signaling cascades that ultimately modulate cell growth, differentiation, and proliferation (Hannun and Obeid, 2008). *S. schenckii* mycelia synthesize only GlcCer, while yeast forms are found to contain both GlcCer and GalCer, suggesting that the ceramide galactosyltransferase may be activated during the *S. schenckii* mycelium-yeast switch or inhibited during the yeast-to-hypha transition (Dickson and Lester, 1999; Toledo et al., 2000). Structural analyses of neutral GSLs between *A*. *fumigatus* and *S. schenckii* show that GalCer and GlcCer possess identical ceramide backbones, except that GalCer contains a higher proportion of Δ3-unsaturated FA (Toledo et al., 1999, 2000). Similarly, the GalCer production and differential Δ3-unsaturation of FAs may constitute a molecular mechanism of GSL control over fungal morphogenesis through the activation/inactivation of signal transduction pathways (Fernandes et al., 2018).

## The Potential Exploitation and Perspective of Filamentous Fungal GlcCer and GalCer

Acidic GSLs in plants and fungi are more structurally diverse and difficult to analyze due to unavailability of commercial standards required by mass spectrometry. Therefore, neutral GSLs are mainly exploited and applicated in the cosmetics and health food products. Neutral GSLs are a common natural component in plants, animals, and fungi. Experiments show that neutral GSLs are not toxic, and no adverse reactions in humans. Therefore, neutral GSLs, mainly GlcCers obtained from plants, are now widely added in cosmetics as moisturizing ingredients and health foods as functional component (Alessandrini et al., 2004; Tessema et al., 2017).

### GlcCers Are Important for the Barrier Function of Skin as Moisturizing Ingredients or Dietary Supplements

**FIGURE 4:**
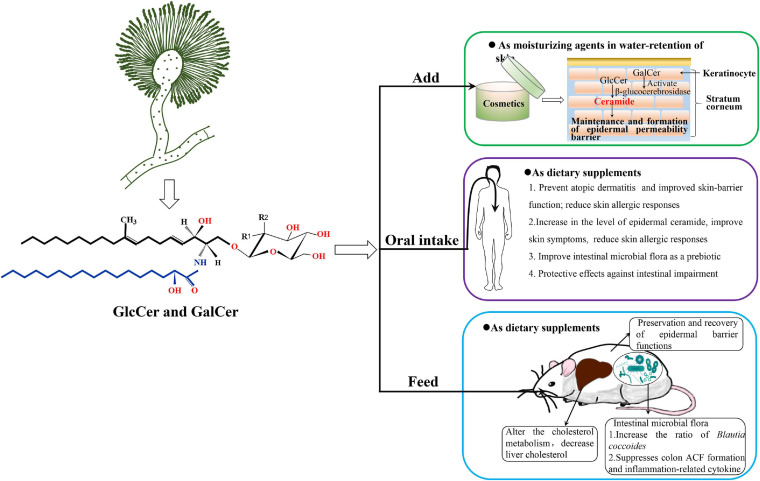
The potential application diagram of filamentous fungal glycosylceramides, taking *A*. *oryzae* as an example. *A*. *oryzae* produces GlcCers and GalCers of glycosylceramides which might be safe to add in cosmetics as moisturizing ingredient or health food products as nutritional supplements. *A*. *oryzae* glycosylceramides, which function as a prebiotic, have been confirmed to increase ratio of *Blautia coccoides* and significantly decrease liver cholesterol in obese mice fed with koji glycosylceramide. Monosaccharide may be either glucosyl (Glc) (R1 = OH, R2 = H) or galactosyl (Gal) residues (R1 = H, R2 = OH) in the chemical structure.

Ceramide is thought to be a critical molecule in the maintenance and formation of the epidermal permeability barrier and an important component in water-retention of skin (Hamanaka et al., 2005). GlcCer can be hydrolyzed to ceramide by β-glucocerebrosidase in keratinocytes, and the resultant ceramide is stored in stratum corneum (Figure 4). Levels of GlcCer significantly increase during epidermal differentiation, and then GlcCer is enzymatically hydrolyzed to ceramides to regulate permeability barrier function. The level of epidermal ceramide is regulated by a balance between β-glucocerebrosidase, sphingomyelinase, and ceramidase (Holleran et al., 2006). A deficiency or inhibition of β-glucocerebrosidase in the epidermis can alter the distribution of ceramide and GlcCer, resulting in decreased the epidermal permeability barrier function (Alessandrini et al., 2004; Carneiro et al., 2011). Low levels of GluCer and ceramide in atherosclerosis-prone mice led to skin inflammation and hair discoloration and loss (Bedja et al., 2018). Prior studies demonstrated that all molecular species of stratum corneum ceramides are derived from GlcCer (Uchida et al., 2000; Hamanaka et al., 2002). In addition, GlcCer also prevents dehydration of the stratum corneum and repaired the barrier function of skin. Therefore, the GlcCers play critical role in enhancing the epidermal permeability barrier function.

Furthermore, the beneficial effects of oral intake of plant GlcCer for skin hydration and skin barrier reinforcement have also been established in several studies involving animal models (Tsuji et al., 2006; Uchiyama et al., 2008; Yeom et al., 2012; Kawano and Umemura, 2013) as well as human subjects (Miyanishi et al., 2005; Kimata, 2006; Uchiyama et al., 2008; Guillou et al., 2011; Figure 4). The present studies suggest that the lipophilic fraction of GlcCer, present in plants has protective effects against intestinal impairment, but it requires extraction since digestion alone is not enough to elicit its complete protective action. Therefore, purified GlcCers are usually added in health food as a function complement. Some studies report that an intake of 0.6–1.8 mg/day of GlcCer from supplements is enough to improve human skin health (Uchiyama et al., 2008; Hirakawa et al., 2013). Shimoda et al. (2012) established the effects of GlcCer on the changes in epidermal ceramide and GlcCer in mice after oral intake of rice-derived GlcCer, as well as in a human epidermal equivalent. These findings demonstrated an increase in the level of epidermal ceramide follow by a decrease in the amounts of GlcCer (accompanied with enhanced β-glucocerebrosidase and GlcCer synthase expressions), suggesting epidermal GlcCer metabolism enhancing effect by oral intake of GlcCers. Oral administration of GluCer from maize significantly reduced UVA-induced wrinkle formation in the skin as well as epidermal hypertrophy of hairless mice (Shimada et al., 2011). Oral intake of konjac GlcCer has been reported to decrease the transepidermal water loss (TEWL) in atopic dermatitis (AD) patients (Miyanishi et al., 2005), and improve skin symptoms (including TEWL reduction) and reduce skin allergic responses in children with moderate AD (Kimata, 2006). These studies support the beneficial effects of oral intake of plant GlcCer and their potential complementary and alternative therapeutic applications in the restoration and maintenance of skin barrier function. Therefore, GlcCers exhibit a wide potential for development and application as an important biological resource in improving epidermal barrier function.

### GlcCers Are Important in Repairing Intestinal Impairments as Functional Components

In addition to prevent AD and improved skin-barrier function, plant GlcCer also prevent intestinal impairment as dietary supplements (Figure 4). Recently, the incidence rate of intestinal impairments, such as colon cancer and inflammatory bowel disease (IBD), has increased in East Asian countries and Western countries (Molodecky et al., 2012). It is difficult to recover completely from IBD and these patients have an increased risk of developing colon cancer (Triantafillidis et al., 2009). There are many studies on the effect of GlcCer contained in food on intestinal impairment. *In vitro* experiments indicate the possibility that GlcCer protects the colon surface from the harmful effects of various drugs (Yamashita et al., 2017). In addition, GlcCer has been shown to have an apoptosis-inducing effect on colon cancer cells *in vitro* (Aida et al., 2004). Previous studies demonstrate that uptake of dietary GlcCer may improve the microenvironment of intestinal tract *via* modulating the intestinal microbiota (Schmelz et al., 2015). Yamashita et al. (2019) had confirmed that dietary GlcCer can significantly suppress aberrant crypt foci (ACF) formation and the production of inflammation-related cytokines in mice fed with rice-derived extracted GlcCer, which suggest GlcCer extracted from polished rice has protective effects against intestinal impairment. Collectively, dietary GlcCers can be digested by the intestinal microbial flora and display preventive and chemotherapeutic effects on colon cancer in animal models; however, clinical trials are urgently needed to investigate the response of colon carcinogenesis to dietary GlcCer in the future.

### The Application of Fungal GlcCer and GalCer in Cosmetics or Health Foods

Although there are few studies on the applications of fungal glycosylceramides, we summarized several applications of them in cosmetics or foods. Interestingly, Takahashi confirmed that GlcCer produced by *A*. *oryzae* constituted the most abundant species (43% of the total GlcCer) in the sake lees, which is brewed with *A*. *oryzae* and *S. cerevisiae*, and has long been recognized for its moisture-holding activity in cosmetics in Japan (Takahashi et al., 2014). Torula yeast (*Candida utilis*)-derived GlcCer has been reported to increase dermal fibroblast proliferation and collagen production to maintain dermal elasticity (Fukunaga et al., 2019). Miyagawa et al. (2019) investigated the effects of glycosylceramides on gene expression in normal human epidermal keratinocytes, which reveal that *koji* and *Aspergillus luchuensis* and *A*. *oryzae* glycosylceramides increased the expression of occludin (OCLN, an epidermal tight junction protein) and ATP-binding cassette sub-family A member 12 (ABCA12, a cellular membrane transporter) to increase ceramide in the keratinocytes. These results indicated that glycosylceramides have an effect of increasing genes expression which involved in skin barrier function and the transport of lipids in the keratinocytes, and suggest that *koji* exerts its cosmetic effect by increasing ceramide and tight junctions *via* glycosylceramides. In addition to function in cosmetics, fungal neutral GSLs also play an important role in foods. For example, Ferdouse et al. (2018) have proved that addition of *A*. *oryzae* glycosylceramide can affect the flavor and metabolic profiles of sake yeast in the manufacture of sake. They also demonstrate that addition of *A*. *oryzae*, *Glycine max*, and *Grifola frondosa* GlcCers confer a similar effect on the flavor profiles of sake yeast (Ferdouse et al., 2018), which indicate that the effects of plant GlcCer and *A*. *oryzae* GlcCer on flavor profiles are similar. In addition, *A*. *oryzae* glycosylceramide, used as a prebiotic in obese mice, can be digested by the intestinal microbial flora and increased ratio of *B. coccoides*, which is a potentially beneficial species in the intestine (Hamajima et al., 2019). Besides, feeding of *A*. *oryzae* glycosylceramide to obese mice can alter the cholesterol metabolism that liver cholesterol is significantly decreased in obese mice fed with *A*. *oryzae* glycosylceramide (Figure 4). These results will be of value in the utilization of fungal GlcCer and GalCer for cosmetics and functional foods.

### The Limitations of Plant GlcCer and Animal GalCer in Application

Although neutral GSLs are originally derived from soybean and bovine sources, currently almost all the GlcCers used in cosmetics or health foods are extracted from plants. GlcCers from wheat flour (Ohnishi et al., 1985), potatoes (Bartke et al., 2006), maize, rice (Sugawara et al., 2010), and konjac (Uchiyama et al., 2008; Usuki et al., 2015) are now widely added in cosmetics as moisturizing agents or in health foods as “functional components.” The most abundant classes of GSLs in plant tissue are mono-GlcCers. In contrast, the GalCer of glycosylceramides is rarely detected or reported in plants (Sullards et al., 2000; Spassieva and Hille, 2003). In cosmetics, GlcCer must be hydrolyzed to ceramide that is then deposited in stratum corneum to form the epidermal barrier. The more selective and efficient approach of transforming GlcCer to ceramide is enzymatic hydrolysis of β-glucocerebrosidase. Interestingly, GalCer, restricted to mainly in neural tissues in mammals, can activate β-glucocerebrosidase to hydrolyze GlcCer in keratinocytes and increase ceramide content to improve dry skin and AD (Alessandrini et al., 2004). However, GalCer is mainly extracted from neural tissues of animals such as cows and has been for the subject of several studies until the discovery of bovine spongiform encephalopathy. Although the animal glycosylceramides obtained from bovine brain and biotechnological sources have been investigated, the safety profile for cosmetic and food applications has not yet been established (Adam, 2001; Ono et al., 2010). Thus, GalCer from neural tissues of animals is not acceptable for cosmetic or other human use due to the underlying risk for prion diseases. As an alternative, glycosylceramides isolated from edible plants are highly safe and preferable for cosmetic and therapeutic applications. Unfortunately, plant glycosylceramides only contain GlcCer and are totally devoid of GalCer. Therefore, it is important to seek new biological resources that can synthesize both GlcCer and GalCer, along with attempts to improve their contents by modifying the biosynthetic pathway of neutral GSLs using genetic engineering technology in known resources.

### The Advantage of Exploiting GlcCer and GalCer From Filamentous Fungi Aspergillus

Glycosylceramides, which have now been commercialized as moisturizing agents or dietary supplements for dry skin, are mainly sourced from edible plants that only contain GlcCer and are completely without GalCer. Surprisingly, there are many reports that filamentous fungi *Aspergillus* species, such as *A*. *oryzae*, *A*. *awamori*, and *A*. *sojae* that are used in various Japanese fermented foods and drinks, can produce both GlcCer and GalCer (Fujino and Ohnishi, 1977; Zhang et al., 2007; Hirata et al., 2012; Tani et al., 2014). Therefore, glycosylceramides from *Aspergillus* that include both GlcCer and GalCer can make up for the lack of GalCer in plants and would be more effective for their barrier function of skin in cosmetics as compared to plant GlcCer, due to the GalCer can activate β-glucocerebrosidase to promote the hydrolysis of GlcCer to ceramide (Figure 4; Alessandrini et al., 2004; Tani et al., 2014). Furthermore, previous studies have elucidated that koji (*A*. *oryzae*, *A*. *awamori*, and *A*. *sojae*) contains abundant glycosylceramides (0.5–2 mg/g dry weight koji), which are comprised of GalCer (30.3%) and GlcCer (69.7%), and are one of the highest amounts found in any cuisine (Hirata et al., 2012; Hamajima et al., 2016). Therefore, since filamentous fungi *Aspergillus* are economically important in the industrial production and can synthesize both GlcCer and GalCer, they would be new sources for exploiting neutral glycosylceramides in future.

Another advantage of filamentous fungi in the production of GlcCer and GalCer is that the cell growth cycle of fungi is much shorter than that of plants, which can provide a large amount of mycelium for extraction of GlcCer and GalCer. In addition, most filamentous fungi used in the production of fermented foods are safe for humans, especially *A*. *oryzae*, which is generally regarded as safe according to the FDA and has been used for making fermented foods such as saké, shoyu (soy sauce), and miso (soybean paste) for thousands of years. Therefore, the *A*. *oryzae* would be a suitable source for production of GlcCer and GalCer due to their food safety. Furthermore, the availability of genome sequences of *A*. *oryzae* and the availability of DNA arrays, GeneChips, and RNA sequence have provided an unprecedented resource for studying and modifying the biosynthetic pathways of GlcCer and GalCer. In summary, we believe that filamentous fungi would be important resources for exploiting neutral glycosylceramides in the future, especially GalCer.

## Conclusion and Future Trends

There is no doubt that exogenous or endogenous GSLs as moisturizing ingredients or dietary supplements continue to surprise us today and there are exciting times for the field. GSLs are a chemically complex group of substances, widely existing in fungi, plant, and animals. In this review, we summarized the kinds and structures of filamentous fungi GSLs. The synthetic pathway and biological roles of GSLs in filamentous fungi were also discussed. Importantly, we particularly focused on the important role of neutral GSLs adding in cosmetics as moisturizing ingredients and dietary supplements as functional components. Meanwhile we also drawn attention to the limitation of GSLs from plants and animals and the advantage of GSLs from filamentous fungi. Collectively, neutral GSLs from filamentous fungi *Aspergillus* will play important roles in the barrier function of skin and intestinal impairment of human health, which greatly increase the demand for neutral GSL. Nowadays, the market demand of GSLs as a new raw ingredient in beauty products is increasing rapidly at an annual growth rate of about 15%.

Since neutral GSLs are benefit to human health, a broader assessment of the types of GSLs adding in cosmetics and health foods is needed because they may have beneficial effects on human health. Not only are endogenous GSLs involved in obesity-related pathologies, prevention or treatment of obesity, and reducing skin allergic responses, but exogenous GSLs may be beneficial in the barrier function of skin (Walls et al., 2013; Miyagawa et al., 2019; Le Barz et al., 2020). Nowadays neutral GSLs, mainly GlcCers obtained from plants, are now widely included in cosmetics or health food. Previous studies done on improving AD and skin moisture suggest that oral intake of GlcCer activates enteric canal immunity and ceramide metabolism in the skin, rather than the direct reutilization of dietary GlcCer (Ono et al., 2010; Duan et al., 2012), which conclude that the maintenance of intestinal homeostasis by dietary GlcCer might be indirectly related to these mechanisms. In addition, GlcCers mast be hydrolyzed by β-glucocerebrosidase to ceramide in keratinocytes or mucosal cells of the small intestine and colon; and then ceramide is taken up directly by intestinal cells in human intestinal epithelial cell models (Nicolas et al., 2005). GalCer was reported to activate β-glucocerebrosidase in keratinocytes and increase ceramide content to improve dry skin and prevent AD (Alessandrini et al., 2004; Carneiro et al., 2011). Unfortunately, neutral GSLs derive from plants without any GalCer; while GalCer extracted from neural tissue of animals such as bovine has been applied for several research until the finding of bovine spongiform encephalopathy. What is more, the production of GalCer from neural tissues of animals would not be acceptable for cosmetic or other human uses. Also, GalCer from pathogenic fungi is not suitable for oral intake. Since neutral GSLs from plants do not contain GalCer, and GalCer from animals is at risk of viral infection, those from filamentous fungi *Aspergillus* that include both GlcCer and GalCer would be highly effective for barrier function of skin and safe for health food as dietary supplements. Therefore, the filamentous fungi *Aspergillus*, synthesizing both GlcCer and GalCer, could be an amenable source to produce glycosylceramides, because they are food safe. We believe that the development of GlcCer and GalCer, especially GalCer, from *Aspergillus* would become a trend and major source in the future.

## Author Contributions

JG: conceptualization and writing—original draft preparation. CJ: writing—review and editing, conceptualization, and funding acquisition. BZ: supervision and funding acquisition. BH: project administration. All authors contributed to the article and approved the submitted version.

## Conflict of Interest

The authors declare that the research was conducted in the absence of any commercial or financial relationships that could be construed as a potential conflict of interest.
